# Novel missense mutation in *PTPN22* in a Chinese pedigree with Hashimoto’s thyroiditis

**DOI:** 10.1186/s12902-018-0305-8

**Published:** 2018-11-01

**Authors:** Licheng Gong, Beihong Liu, Jing Wang, Hong Pan, Anhui Qi, Siyang Zhang, Jinyi Wu, Ping Yang, Binbin Wang

**Affiliations:** 1 Department of Cardiology, China Japan Union Hospital of Jilin University, Chang Chun, Jilin, 130000 China; 2 Graduate School of Peking Union Medical College, Beijing, China; 3 Center for Genetics, National Research Institute of Family Planning, Beijing, China; 4 Department of Medical Genetics and Developmental Biology, School of Basic Medical Sciences, Capital Medical University, Beijing, China; 5 National Research Institute for Family Planning, 12 Dahuisi Road, Haidian, Beijing, 100081 China

**Keywords:** Hashimoto’s thyroiditis, Whole-exome sequencing, *PTPN22*, Mutation

## Abstract

**Background:**

Hashimoto’s thyroiditis is a complex autoimmune thyroid disease, the onset of which is associated with environmental exposures and specific susceptibility genes. Its incidence in females is higher than its incidence in males. Thus far, although some susceptibility loci have been elaborated, including *PTPN22*, *FOXP3*, and *CD25*, the aetiology and pathogenesis of Hashimoto’s thyroiditis remains unclear.

**Methods:**

Four affected members from a Chinese family with Hashimoto’s thyroiditis were selected for whole-exome sequencing. Missense, nonsense, frameshift, or splicing-site variants shared by all affected members were identified after frequency filtering against public and internal exome databases. Segregation analysis was performed by Sanger sequencing among all members with available DNA.

**Results:**

We identified a missense mutation in *PTPN22* (NM_015967.5; c. 77A > G; p.Asn26Ser) using whole-exome sequencing. *PTPN22* is a known susceptibility gene associated with increased risks of multiple autoimmune diseases. Cosegregation analysis confirmed that all patients in this family, all of whom were female, carried the mutation. All public and private databases showed that the missense mutation was extremely rare.

**Conclusions:**

We found a missense mutation in *PTPN22* in a Chinese HT pedigree using whole-exome sequencing. Our study, for the first time, linked a rare variant of *PTPN22* to Hashimoto’s thyroiditis, providing further evidence of the disease-causing or susceptibility role of *PTPN22* in autoimmune thyroid disease. Functional studies regarding the effects of this variant on thyroid autoimmunity and thyroid function are warranted.

## Background

Autoimmune thyroid disease (AITD) constitutes a complex class of diseases, including Graves’ disease (GD) and Hashimoto’s thyroiditis (HT); these are associated with interactions of specific susceptibility genes and environmental exposures [1, 2]. Both GD, manifested as hyperthyroidism, and HT, manifested as hypothyroidism, exhibit common characteristics of the production of thyroid autoantibodies and the invasion of thyroid lymphocytes. AITD is one of the most prevalent autoimmune diseases, affecting approximately 5% of the general population [1, 2]. The incidence of GD and HT demonstrates a significant genetic effect in populations with adequate iodine intake in different geographical locations, because these populations encounter different environmental factors [3]. Approximately 37% of families with AITD exhibit either of these two disorders [4]. In the Whickham survey, the prevalence of spontaneous hypothyroidism was 1.5% in females—far higher than the 0.1% observed in males [5]. Thus far, the aetiology and pathogenesis of AITD remain unclear. Some scholars have speculated that separate genetic factors are related to the onset of GD and HT, while others support the concept of common genetic susceptibility factors [4].

Previous studies advancing the understanding of the genetic aetiology of AITD have expanded the field of thyroid autoimmunity. Although some susceptibility loci associated with AITD have been elaborated in past reports, the following correlations with AITD may prove more conclusive: *PTPN22*/1q13.2 (T lymphocyte signalling), *TSHR*/14q31.1 (thyrotropin receptor), *HLA*/6p21 (human leukocyte antigen) and *CTLA4*/2q33.2 (T-regulatory cell function) [6]. *TSHR* has been confirmed as a disease-specific locus by GWAS and other association studies [7]. The disease-predisposing genotype (TT) of SNP rs12101261 has been associated with reduced thymic expression of *TSHR* mRNA [7]. Reduction in thymic expression of *TSHR* may contribute to sustained escape of pathogenic T cells from the centre and an increased risk of *TSHR* autoimmunity. *FOXP3* and *CD25*, in connection with AITD, play a key role in the establishment of peripheral tolerance [8]. The *DXS573* microsatellite in *FOXP3* linkage disequilibrium (LD) is associated with AITD in Caucasian female AITD patients [9]. An A/C polymorphism at − 3279 has been associated with the development of therapeutic resistance in patients with GD, whereas the CC genotype at − 2383 has been associated with severe HT [10]. Taken together, these prior studies demonstrate that the *FOXP3* polymorphism is associated with AITD. Several other susceptible loci have also been associated with AITD, including *FCRL3* (Fc receptor-like 3), *RNASET2* (ribonuclease T2), *SCGB3A2* (secretoglobin, family 3A member 2) [6, 11–14].

Previous studies have shown that *PTPT22* is associated with a variety of immune diseases [15]. Jacobson et al. [16] reported that the R620W SNP was related to the occurrence of HT. However, some reports have indicated a negative correlation between R620W and AITD [17, 18], while other reports have indicated a positive correlation [19–21]; this dispute remains unresolved.

Notably, unrelated individuals have been the primary focus in past research studies; there are few pedigree studies, and it is difficult to establish whether HT is an inherited disease. Here, we report a Chinese HT pedigree with six affected members who exhibit an autosomal-dominant inheritance pattern. Whole-exome sequencing (WES) revealed that a rare heterozygous missense variant in *PTPN22* co-segregated with this disorder. This mutation was identified for the first time in patients with HT.

## Methods

### Participants

**Fig. 1:**
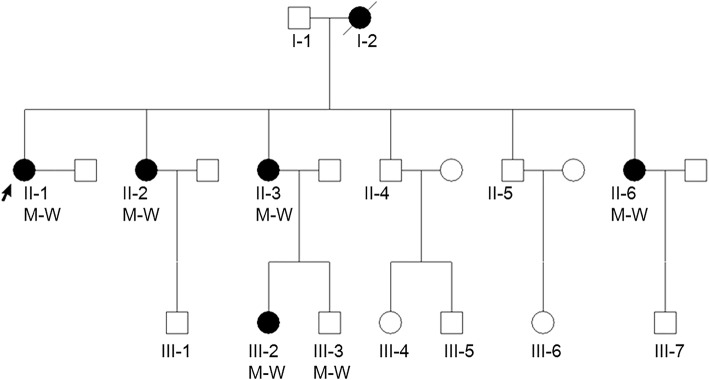
Pedigree chart of the family with *PTPN22* missense mutation in this study, along with the cosegregation pattern of the heterozygous mutations in *PTPN22*

We examined a Chinese family in which six individuals across three generations exhibit hypothyroidism; all patients are females (Fig. 1). We collected peripheral blood samples from one unaffected and five affected individuals in this family, as well as results of thyroid function tests, thyroid antibody tests, rheumatoid factor and thyroid colour Doppler ultrasonography. This research was approved by the ethical committees of the China Japan Union Hospital of Jilin University. Written informed consent was obtained from all study participants.

### Clinical examinations

Diagnosis of HT was made on the following basis: clinical and biochemical evidence of hypothyroidism requiring thyroxine replacement therapy, as well as increased expression of autoantibodies to thyroid peroxidase (TPO) and antithyroglobulin, as measured by specific radioimmunoassay. Each participant was interviewed and signed a letter of consent before inclusion in the study.

### WES and validation

DNA was extracted from peripheral venous blood by using the QIAamp DNA Blood Mini Kit (QIAGEN, Hilden, Germany). Exons of DNA samples were captured with the SureSelect Human All Exon V5 (Agilent, Santa Clara, CA, USA) and then sequenced on the Illumina Hiseq4000 sequencer (Illumina, Saint Diego, CA, USA). By using BWA − 0.7.10 (the Burrows-Wheeler Alignment Tool), the reads were mapped to the human reference genome (hg19). Variants including single nucleotide variants (SNVs) and indels were called by using GATK 3.v4 (Genome Analysis Toolkit) and annotated with SnpEff _v4.1.

### Segregation analysis

Sanger sequencing was used to perform co-segregation analysis of c. 77A > G of *PTPN22* among pedigree members with available DNA. The primers were as follows: forward, 5**′**- GTTCATTTGGGACATAAGG-3**′**; reverse, 5**′**- CCAGGAGTTCAAGGCTAC-3**′**.

## Results

### Clinical features

This family exhibited HT with unique clinical characteristics. Over three generations, six family members were affected, and all affected individuals were female. The proband (II-1) was a 58-year-old woman who was admitted to the hospital for review of thyroid disease. She had exhibited thyroiditis for 3 years and had been tested for thyroid nodule and diffuse thyroid changes. Thyroid function tests showed that she had an elevated thyroid peroxidase autoantibody (TPOAb) level (244 IU/ml, normal range: 0–34.0 IU/ml) and an elevated thyroid stimulating hormone (TSH) level (5.37 mIU/L, normal range: 0.372–4.94 mIU/L). Therefore, she was diagnosed with HT. Her two sisters and a niece also exhibit thyroid nodules and diffuse thyroid changes; all affected members’ results of thyroid function tests were similar to those of the proband, with abnormally high values of TPOAb. Additionally, II-6 also had rheumatoid arthritis with a high serum level of rheumatoid factor (74.7 IU/ml, normal range: 0–20 IU/ml). All patients received different doses of thyroxine replacement therapy after diagnosis. We performed colour Doppler echocardiography, blood routine examination, and biochemical examination for all patients; no secondary systemic diseases caused by hypothyroidism were found. Other family members did not exhibit significant thyroid abnormalities. Clinical examination results are shown in Table 1.

**Table 1:** Characteristics of inspection results

Patients	TPOAb(IU/ml)	TSH(mIU/L)	TgAb	TN	DTC	TRT
II-1	244.0	5.37	N	+	+	Y
II-2	397.8	8.12	N	+	+	Y
II-3	235.5	9.86	N	N	N	Y
II-6	267.8	3.16	N	+	+	Y
III-2	230.3	19.84	N	+	+	Y

When we first contacted the family, all patients had begun thyroxine replacement therapy. The measured values of thyroxine in all patients were in the normal range

*TPOAb* Thyroid peroxidase autoantibody, *TSH* Thyroid-stimulating hormone, *TgAb* Antithyroglobulin autoantibody, *TN* Thyroid nodule, *+* Existence of this change, *N* Refused the examination, *DTC* Diffuse thyroid changes (caused by thyroiditis), *TRT* Thyroxine replacement therapy, *Y* Receiving treatment

### WES sequencing

We selected the proband and her three affected family members (II-2, II-6, III-2) for WES. We focused on missense, nonsense, splice-site, and frameshift mutations that were shared by all sequenced members. Variants with a frequency > 1% in public databases (1000Genomes, ESP6500si, and ExAC Asian population) and an internal database (400 exomes) were discarded. After this filtering had been performed, 18 genes remained, among which we found a rare missense heterozygous variant (NM_015967.5:c.A77G; p.N26S) in *PTPN22*, a known susceptibility gene for HT. In this variant, an A to G substitution occurred at the 77th base, leading to an amino acid change from asparagine to serine at the 22nd residue. This variant was rare in several public databases (0.02% in 1000Genomes, 0.05% in ESP6500, none in ExAC Asian population) and absent from our internal exome database. Therefore, we considered the variant to have a high likelihood of association with familial HT.

### Co-segregation analysis

**Fig. 2:**
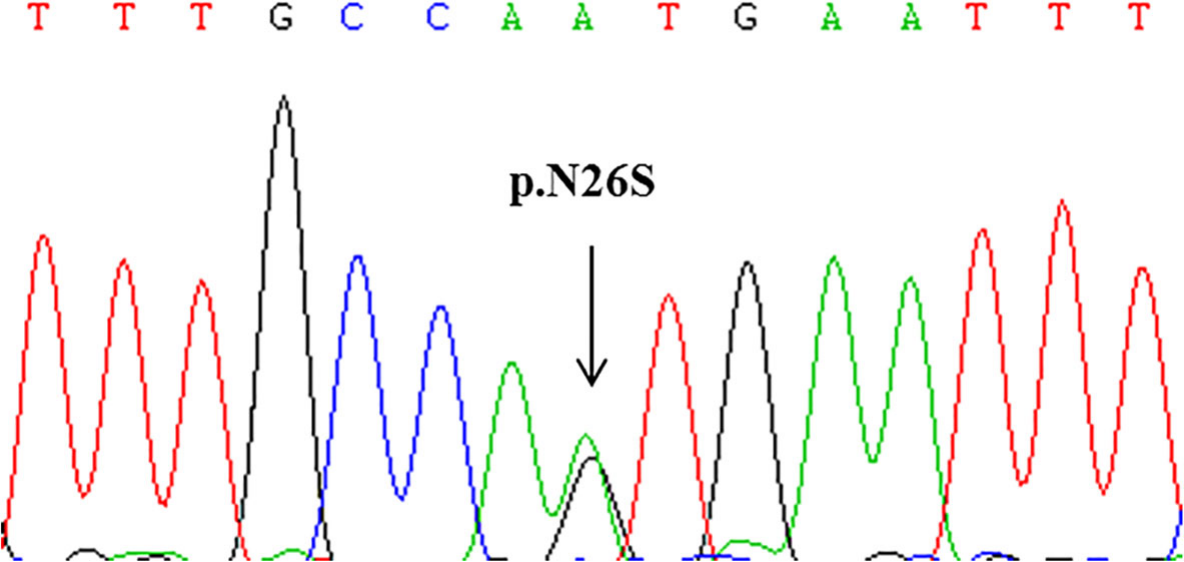
Sanger sequencing results for the missense mutation in *PTPN22*

Co-segregation analysis showed that all affected members carried the variant (Fig. 2). In addition, we found that III-3, a male member with normal health thus far, also carried the variant.

## Discussion

*PTPN22* is located at chromosome 1p13.2 and encodes a tyrosine phosphatase that is expressed by haematopoietic cells and acts as a key regulator of immune homeostasis through inhibition of T-cell receptor signalling and promotion of type I interferon responses [22]. *PTPN22* has been identified as the main susceptible gene for multiple autoimmune diseases, including rheumatoid arthritis [23], juvenile idiopathic arthritis [24], psoriatic arthritis [25], systemic lupus erythematosus [26], systemic sclerosis [27] and some forms of vasculitis [28]. This gene is considered the second-most important predisposing gene for human autoimmune diseases, after *HLA*. Therefore, abnormalities of *PTPN22* can lead to the occurrence of autoimmune diseases.

The *PTPN22* gene encodes lytic tyrosine phosphatase (LYP), which comprises 807 amino acid residues [29]. LYP acts as an inhibitor of T cell activation by binding to variety of signal transduction molecules, such as Csk kinase, which is active in T cell activation. Arginine substitution for tryptophan at codon 620 of the LYP protein (R620W) has been associated with increased risks of rheumatoid arthritis [23, 30] and systemic lupus erythematosus (SLE) [15]. Further studies showed an association between this SNP and AITD, including GD [31] and HT [32]. The *PTPN22* R620W SNP elicits a functional change in LYP, such that the tryptophan-bearing LYP allele cannot bind the C-terminal src kinase (Csk) [33]; this causes proliferation of T cells. Concomitantly, the levels of several Ig isotypes are increased [32]. Among these antibodies, levels of IgG and IgG4 were positively correlated with the titre of anti-thyroperoxidase antibody (anti-TPO) [34]. Changes in level of anti-TPO correlated positively with the development of hypothyroidism and an increased inflammatory reaction [34]. As shown in Table 1, the patients in this study have higher TPO levels than normal, a phenomenon that supports this mechanism. We hypothesized that the mechanism of the missense mutation in *PTPN22* (N26S) is similar to that of R620W SNP.

LYP is an approximately 105-kDa Class I protein tyrosine phosphatase (PTP) [35, 36], which includes a C-terminal PEST-enriched domain and a classical N-terminal protein tyrosine phosphatase catalytic domain; these are separated by an approximately 300-amino acid interdomain [30]. The enzyme includes four putative polyproline motifs (P1-P4) [30]. The missense mutation *PTPN22* (N26S) is located on the classical catalytic domain of the N-terminal protein tyrosine phosphatase; however, little is known regarding its specific function.

The inter domain of LYP harbours protein-protein interaction motifs and putative phosphorylation sites. Liu et al. [30] showed that through a direct intramolecular interaction and inhibition of the catalytic domain, the interdomain plays an important role in regulating catalytic activity of the protein. For example, on the basis of a constitutive interaction between the N-terminal SH2 domain and the catalytic domain, SHP-1 is inhibited; it is released following recruitment of the domain to phosphorylated targets [37]. Changes in the interactions between domains can indirectly mediate the functional effects of protein interactions and/or post-translational modifications located in other parts of the protein. We hypothesize that the R620W SNP affects interactions among domains in LYP, leading to the occurrence of HT. Identification of the specific molecular mechanism requires further research.

In this study, we found that the patients with HT were all females in this family, consistent with the previously reported finding that the incidence of thyroid disease in females is much higher than in males [38]. Prior reports had showed that the preponderance of thyroid autoimmunity in females is most likely due to the influence of sex steroids. Furthermore, the presence of TPO autoantibodies is the strongest risk factor for both hyper- and hypothyroidism; smoking is negatively correlated with the presence of TPO antibodies. Overall, the incidence of HT in females was significantly higher than the incidence of HT in males [39].

In the present study, we found that III-3 carried the missense mutation of *PTPN22* (A77G), but did not exhibit any thyroid disorder. Men are less susceptible to autoimmune disease than women, which may be why III-3 was unaffected. Additionally, the young age of III-3 (24 years of age) may be a contributing factor, because autoimmune diseases occur most frequently between the ages of 45 and 65 [34]. Finally, we hypothesize that *PTPN22* N26S may be similar to the R620W SNP as a risk locus, which can increase the risk of thyroid disease; however, it may not show disease in carriers.

## Conclusions

In summary, we identified a missense mutation in *PTPN22* in a Chinese HT pedigree by using WES. This finding supports follow-up studies regarding HT, potentially facilitating more comprehensive genetic research in HT. Follow-up experiments are needed.

## Abbreviations

AITD: Autoimmune thyroid disease
GD: Graves’ disease
HT: Hashimoto’s thyroiditis
SNVs: Single-nucleotide variants
TPO: Thyroid peroxidase
WES: Whole-exome sequencing
TPOAb: Thyroid peroxidase autoantibody.
TSH: Thyroid-stimulating hormone
LYP: Lytic tyrosine phosphatase
SLE: Systemic lupus erythematosus
Csk: C-terminal src kinase
PTP: Protein tyrosine phosphatase

## References

[1] Jacobson DL, Gange SJ, Rose NR, Graham NM (1997). Epidemiology and estimated population burden of selected autoimmune diseases in the United States. Clin Immunol Immunopathol.

[2] Antonelli A, Ferrari SM, Corrado A, Di Domenicantonio A, Fallahi P (2015). Autoimmune thyroid disorders. Autoimmun Rev.

[3] Tomer Y, Davies TF (2003). Searching for the autoimmune thyroid disease susceptibility genes: from gene mapping to gene function. Endocr Rev.

[4] Tomer Y, Ban Y, Concepcion E, Barbesino G, Villanueva R, Greenberg DA, Davies TF (2003). Common and unique susceptibility loci in graves and Hashimoto diseases: results of whole-genome screening in a data set of 102 multiplex families. Am J Hum Genet.

[5] Tunbridge WM, Evered DC, Hall R, Appleton D, Brewis M, Clark F, Evans JG, Young E, Bird T, Smith PA (1977). The spectrum of thyroid disease in a community: the Whickham survey. Clin Endocrinol.

[6] Chu X, Pan CM, Zhao SX, Liang J, Gao GQ, Zhang XM, Yuan GY, Li CG, Xue LQ, Shen M (2011). A genome-wide association study identifies two new risk loci for Graves’ disease. Nat Genet.

[7] Stefan M, Wei C, Lombardi A, Li CW, Concepcion ES, Inabnet WB, Owen R, Zhang W, Tomer Y (2014). Genetic-epigenetic dysregulation of thymic TSH receptor gene expression triggers thyroid autoimmunity. Proc Natl Acad Sci U S A.

[8] Lee HJ, Li CW, Hammerstad SS, Stefan M, Tomer Y (2015). Immunogenetics of autoimmune thyroid diseases: a comprehensive review. J Autoimmun.

[9] Ban Y, Tozaki T, Tobe T, Ban Y, Jacobson EM, Concepcion ES, Tomer Y (2007). The regulatory T cell gene FOXP3 and genetic susceptibility to thyroid autoimmunity: an association analysis in Caucasian and Japanese cohorts. J Autoimmun.

[10] Inoue N, Watanabe M, Morita M, Tomizawa R, Akamizu T, Tatsumi K, Hidaka Y, Iwatani Y (2010). Association of functional polymorphisms related to the transcriptional level of FOXP3 with prognosis of autoimmune thyroid diseases. Clin Exp Immunol.

[11] Burton PR, Clayton DG, Cardon LR, Craddock N, Deloukas P, Duncanson A, Kwiatkowski DP, MI MC, Wellcome Trust Case Control C, Australo-Anglo-American spondylitis C (2007). Association scan of 14,500 nonsynonymous SNPs in four diseases identifies autoimmunity variants. Nat Genet.

[12] Kochi Y, Yamada R, Suzuki A, Harley JB, Shirasawa S, Sawada T, Bae SC, Tokuhiro S, Chang X, Sekine A (2005). A functional variant in FCRL3, encoding fc receptor-like 3, is associated with rheumatoid arthritis and several autoimmunities. Nat Genet.

[13] Simmonds MJ, Yesmin K, Newby PR, Brand OJ, Franklyn JA, Gough SC (2010). Confirmation of association of chromosome 5q31-33 with United Kingdom Caucasian Graves’ disease. Thyroid.

[14] Song HD, Liang J, Shi JY, Zhao SX, Liu Z, Zhao JJ, Peng YD, Gao GQ, Tao J, Pan CM (2009). Functional SNPs in the SCGB3A2 promoter are associated with susceptibility to Graves’ disease. Hum Mol Genet.

[15] Kyogoku C, Langefeld CD, Ortmann WA, Lee A, Selby S, Carlton VE, Chang M, Ramos P, Baechler EC, Batliwalla FM (2004). Genetic association of the R620W polymorphism of protein tyrosine phosphatase PTPN22 with human SLE. Am J Hum Genet.

[16] Jacobson EM, Tomer Y (2007). The CD40, CTLA-4, thyroglobulin, TSH receptor, and PTPN22 gene quintet and its contribution to thyroid autoimmunity: back to the future. J Autoimmun.

[17] Kahles H, Ramos-Lopez E, Lange B, Zwermann O, Reincke M, Badenhoop K (2005). Sex-specific association of PTPN22 1858T with type 1 diabetes but not with Hashimoto's thyroiditis or Addison's disease in the German population. Eur J Endocrinol.

[18] Ban Y, Tozaki T, Taniyama M, Tomita M, Ban Y (2005). The codon 620 single nucleotide polymorphism of the protein tyrosine phosphatase-22 gene does not contribute to autoimmune thyroid disease susceptibility in the Japanese. Thyroid.

[19] Gu LQ, Zhu W, Zhao SX, Zhao L, Zhang MJ, Cui B, Song HD, Ning G, Zhao YJ (2010). Clinical associations of the genetic variants of CTLA-4, Tg, TSHR, PTPN22, PTPN12 and FCRL3 in patients with Graves’ disease. Clin Endocrinol.

[20] Zhebrun D, Kudryashova Y, Babenko A, Maslyansky A, Kunitskaya N, Popcova D, Klushina A, Grineva E, Kostareva A, Shlyakhto E (2011). Association of PTPN22 1858T/T genotype with type 1 diabetes, Graves’ disease but not with rheumatoid arthritis in Russian population. Aging.

[21] Heward JM, Brand OJ, Barrett JC, Carr-Smith JD, Franklyn JA, Gough SC (2007). Association of PTPN22 haplotypes with Graves’ disease. J Clin Endocrinol Metab.

[22] Cloutier JF, Veillette A (1999). Cooperative inhibition of T-cell antigen receptor signaling by a complex between a kinase and a phosphatase. J Exp Med.

[23] Begovich AB, Carlton VE, Honigberg LA, Schrodi SJ, Chokkalingam AP, Alexander HC, Ardlie KG, Huang Q, Smith AM, Spoerke JM (2004). A missense single-nucleotide polymorphism in a gene encoding a protein tyrosine phosphatase (PTPN22) is associated with rheumatoid arthritis. Am J Hum Genet.

[24] Viken MK, Amundsen SS, Kvien TK, Boberg KM, Gilboe IM, Lilleby V, Sollid LM, Forre OT, Thorsby E, Smerdel A (2005). Association analysis of the 1858C>T polymorphism in the PTPN22 gene in juvenile idiopathic arthritis and other autoimmune diseases. Genes Immun.

[25] Juneblad K, Johansson M, Rantapaa-Dahlqvist S, Alenius GM (2011). Association between the PTPN22 +1858 C/T polymorphism and psoriatic arthritis. Arthritis Res Ther.

[26] Namjou B, Kim-Howard X, Sun C, Adler A, Chung SA, Kaufman KM, Kelly JA, Glenn SB, Guthridge JM, Scofield RH (2013). PTPN22 association in systemic lupus erythematosus (SLE) with respect to individual ancestry and clinical sub-phenotypes. PLoS One.

[27] Diaz-Gallo LM, Gourh P, Broen J, Simeon C, Fonollosa V, Ortego-Centeno N, Agarwal S, Vonk MC, Coenen M, Riemekasten G (2011). Analysis of the influence of PTPN22 gene polymorphisms in systemic sclerosis. Ann Rheum Dis.

[28] Martorana D, Maritati F, Malerba G, Bonatti F, Alberici F, Oliva E, Sebastio P, Manenti L, Brugnano R, Catanoso MG (2012). PTPN22 R620W polymorphism in the ANCA-associated vasculitides. Rheumatology.

[29] Plenge RM, Padyukov L, Remmers EF, Purcell S, Lee AT, Karlson EW, Wolfe F, Kastner DL, Alfredsson L, Altshuler D (2005). Replication of putative candidate-gene associations with rheumatoid arthritis in >4,000 samples from North America and Sweden: association of susceptibility with PTPN22, CTLA4, and PADI4. Am J Hum Genet.

[30] Liu Y, Stanford SM, Jog SP, Fiorillo E, Orru V, Comai L, Bottini N (2009). Regulation of lymphoid tyrosine phosphatase activity: inhibition of the catalytic domain by the proximal interdomain. Biochemistry.

[31] Velaga MR, Wilson V, Jennings CE, Owen CJ, Herington S, Donaldson PT, Ball SG, James RA, Quinton R, Perros P (2004). The codon 620 tryptophan allele of the lymphoid tyrosine phosphatase (LYP) gene is a major determinant of Graves’ disease. J Clin Endocrinol Metab.

[32] Criswell LA, Pfeiffer KA, Lum RF, Gonzales B, Novitzke J, Kern M, Moser KL, Begovich AB, Carlton VE, Li W (2005). Analysis of families in the multiple autoimmune disease genetics consortium (MADGC) collection: the PTPN22 620W allele associates with multiple autoimmune phenotypes. Am J Hum Genet.

[33] Bottini N, Musumeci L, Alonso A, Rahmouni S, Nika K, Rostamkhani M, MacMurray J, Meloni GF, Lucarelli P, Pellecchia M (2004). A functional variant of lymphoid tyrosine phosphatase is associated with type I diabetes. Nat Genet.

[34] Pyzik A, Grywalska E, Matyjaszek-Matuszek B, Rolinski J (2015). Immune disorders in Hashimoto's thyroiditis: what do we know so far?. J Immunol Res.

[35] Alonso A, Sasin J, Bottini N, Friedberg I, Friedberg I, Osterman A, Godzik A, Hunter T, Dixon J, Mustelin T (2004). Protein tyrosine phosphatases in the human genome. Cell.

[36] Mustelin T, Alonso A, Bottini N, Huynh H, Rahmouni S, Nika K, Louis-dit-Sully C, Tautz L, Togo SH, Bruckner S (2004). Protein tyrosine phosphatases in T cell physiology. Mol Immunol.

[37] Pei D, Lorenz U, Klingmuller U, Neel BG, Walsh CT (1994). Intramolecular regulation of protein tyrosine phosphatase SH-PTP1: a new function for Src homology 2 domains. Biochemistry.

[38] Alkhateeb A, Marzouka NA, Tashtoush R (2013). Variants in PTPN22 and SMOC2 genes and the risk of thyroid disease in the Jordanian Arab population. Endocrine.

[39] Strieder TG, Prummel MF, Tijssen JG, Endert E, Wiersinga WM (2003). Risk factors for and prevalence of thyroid disorders in a cross-sectional study among healthy female relatives of patients with autoimmune thyroid disease. Clin Endocrinol.

